# Competence of mosquitoes native to the United Kingdom to support replication and transmission of Rift Valley fever virus

**DOI:** 10.1186/s13071-018-2884-7

**Published:** 2018-05-18

**Authors:** Sarah Lumley, Luis M. Hernández-Triana, Daniel L. Horton, Maria Del Mar Fernández de Marco, Jolyon M. Medlock, Roger Hewson, Anthony R. Fooks, Nicholas Johnson

**Affiliations:** 1 Microbiology Services Division, Public Health England, Wiltshire, UK; 2 School of Veterinary Medicine, University of Surrey, Guildford, UK; 3 Wildlife Zoonoses and Vector-borne Diseases Research Group, Animal and Plant Health Agency, Addlestone, Surrey, UK; 4 NIHR Health Protection Research Unit in Emerging and Zoonotic Infections, University of Liverpool, Liverpool, UK; 5 Department of Clinical Infection, Microbiology and Immunology, University of Liverpool, Liverpool, UK

**Keywords:** Rift Valley fever virus, Mosquito, Vector competence, UK, Arbovirus

## Abstract

**Background:**

Rift Valley fever phlebovirus (RVFV) is a mosquito-borne arbovirus causing severe disease in humans and livestock. It is endemic in Africa and spread to the Arabian Peninsula in 2000 raising concerns it could emerge in Europe. The ability of temperate mosquitoes from the United Kingdom (UK) to support replication and transmission of RVFV is unknown.

**Methods:**

In this study, two colonised lines of *Culex pipiens*, wild-caught *Aedes detritus* and *Ae. rusticus* from the UK were infected with pathogenic strains of RVFV to assess their vector competence. Mosquitoes were offered artificial blood-meals containing 10^6^ or 10^7^ plaque forming units (PFU)/ml RVFV, simulating natural peak viraemia in young ruminants, and maintained at 20 °C or 25 °C for up to 21 days. Bodies, legs and saliva were collected and tested for the presence of viral RNA and infectious virus to determine the infection, dissemination and transmission potential.

**Results:**

Across temperatures, doses and strains the average infection, dissemination and transmission rates were: 35, 13 and 5% (*n* = 91) for *Cx. pipiens* (Caldbeck); 23, 14 and 5% (*n* = 138) for *Cx. pipiens* (Brookwood); 36, 28 and 7% (*n* = 118) for *Ae. detritus*. However, despite 35% (*n* = 20) being susceptible to infection, *Ae. rusticus* did not transmit RVFV. Survival of *Aedes* species was negatively affected by maintenance at 25 °C compared to the more representative peak average British summer temperature of 20 °C. Increased mortality was also observed with some species infected with 10^7^ PFU/ml compared to 10^6^ PFU/ml.

**Conclusions:**

It can be concluded that temperate mosquito species present in the UK demonstrate a transmission potential for RVFV in the laboratory but, even at high temperatures, this occurred at low efficiency.

**Electronic supplementary material:**

The online version of this article (10.1186/s13071-018-2884-7) contains supplementary material, which is available to authorized users.

## Background

Rift Valley fever phlebovirus (RVFV; *Phenuiviridae*) is a pathogen of both veterinary and public health importance: causing abortions and mortality in ruminants and disease ranging from febrile illness to encephalitis and haemorrhagic fever in humans [1, 2]. Endemic throughout Africa RVFV was introduced into the Arabian Peninsula in 2000 [3] providing evidence that it could emerge in new regions. The detection of RVFV specific antibodies in ruminants in Turkey [4], further raises concerns that RVFV could emerge in Europe. The virus is maintained in a transmission cycle between mosquito and mammalian hosts. It infects a broad range of mainly tropical mosquito species predominantly within the genera *Aedes* and *Culex*, with detection in over 50 species in the wild and 47 vector-competent species in laboratory studies (reviewed in [5]).

The potential pathways for RVFV introduction into Europe have been identified [6]. However, there is limited information on the ability of temperate mosquito species to transmit RVFV. In endemic regions, floodwater *Aedes* spp. are considered maintenance vectors of RVFV. There is evidence to suggest RVFV can pass transovarially from infected female mosquitoes to their progeny [5, 7]. This has led to competing hypotheses that RVFV survives long inter-epizootic periods within desiccated eggs of Aedinae species [5, 7] or remains active in wildlife populations undetected by human surveillance [8]. After prolonged periods of rainfall huge numbers of adult mosquitoes emerge, of which a small percentage are thought to be infected (reviewed by Lumley et al. [5]). The expansion of the mosquito population is thought to then drive outbreaks of RVF [7, 9].

*Culex* species are considered amplifying vectors during epizootics, spreading RVFV to humans and ruminants. The northern house mosquito, *Culex pipiens*, was the principal vector in the epizootic in Egypt 1977 [2, 10]. This species exists in two morphologically identical forms biotype *pipiens* and *molestus*, and a hybrid of the two [11]. The main vector in the Egyptian epizootic was likely the *molestus* form [11] but was not distinguished from another member of the complex, *Cx. quinquefasciatus* (southern house mosquito). *Culex quinquefasciatus* occupies tropical regions overlapping with *Cx. pipiens* in central/northern Africa and South Africa [12]. Historically biotypes were not distinguished but differential patterns of vector competence occur between both biotypes and hybrids, demonstrated in the Netherlands, USA and Spain [13–15].

There have been 36 mosquito species reported in the UK [16] of which 15 are Aedinae and 4 *Culex*. Based on records of abundance and feeding preference the following have potential as RVFV vectors: *Ae. annulipes*, *Ae. cantans*, *Ae. caspius*, *Ae. cinereus/geminus*, *Ae. detritus*, *Ae. punctor* and *Ae. rusticus* [17, 18]. The four *Culex* species in the UK are *Cx. europaeus*, *Cx. modestus*, *Cx. pipiens* and *Cx. torrentium.* The *Culex pipiens* f. *pipiens* feeds preferentially on birds and infrequently on humans, whilst the *molestus* biotype feeds readily on humans. The hybrid is considered to exhibit intermediary behaviours [11]. Reports of *Cx. pipiens* f. *pipiens* biting humans and livestock are rare. However, an understanding of the ability of *Cx. pipiens* (*sensu lato*) in the UK to transmit RVFV is of relevance due to its abundance, proximity to humans and livestock, homology to known vectors in Africa and the co-occurrence of biotypes and hybrids. To improve our understanding of the ability of temperate mosquito species to transmit RVFV, the vector competence of four mosquito populations derived from the UK were evaluated.

## Methods

### Source of mosquitoes

Mosquitoes used are listed in Table 1, including collection location, habitat and filial generation. A map of collection sites is presented in Additional file 1: Figure S1. Species were identified using published keys [19, 20] and a polymerase chain reaction (PCR) targeting mitochondrial cytochrome *c* oxidase subunit 1 (*cox*1) gene [21]. Four *Aedes* species were collected from two diverse habitat types during 2016 and 2017. *Aedes rusticus* and *Ae. cantans/annulipes* were collected from woodland frequented by humans and inhabited by deer and cattle. *Aedes detritus* and *Ae. caspius* were collected from two coastal areas with frequent reports of nuisance biting. Two colonised lines within the *Culex pipiens* complex were maintained, previously characterised as a *Cx. pipiens* f. *pipiens* (Caldbeck: CBK) and *Cx. pipiens*/*molestus* hybrid (Brookwood: BKW) (donated by the Pirbright Institute, UK) [22]. Colonised larvae were reared at 22.5 ± 3.5 °C in filtered water and wild-caught juveniles reared at 20 ± 1.5 °C) in collection site water. Both were fed fish flake (Aquarian, Grantham, UK). Colonised adults were maintained at 25 ± 2.7 °C and wild-caught at 20 ± 2.5 °C). Both were maintained on cotton wool saturated in 10% sucrose, with a photoperiod of 12:12 (light: dark) hours. Colonised mosquitoes were blood-fed twice a week on defibrinated horse blood (TCS Biosciences, Buckingham, UK) by hemotek with a parafilm (Sigma-Aldrich, Gillingham, UK) membrane. Black cups were supplied containing filtered water for oviposition and eggs collected and transferred to rearing trays twice a week.

**Table 1:** Provenance of mosquitoes used in Rift Valley fever virus vector competence studies

Species (shorthand)	Location (Reference)	GPS coordinates (DMS)	Origin	Generation	Collection method	Habitat
*Cx. pipiens* Caldbeck (CBK)	Caldbeck, Worcester Park, London	51°22'51.24"N, 0°14'20.399"W	Larvae, pupae (colonised)	> F100^a^	^c^	Freshwater pond/drainage ditch
*Cx. pipiens* Brookwood (BKW)	Brookwood, Surrey	51°18'23.4"N, 0°37'58.08"W	Larvae, pupae (colonised)	> F100^b^	^c^	Container
*Cx. pipiens* (*sensu lato*), *Cx. modestus*	Cliffe Pools, Kent	51°28'21.032"N, 0°28'31.575"E	Larvae, pupae	F0	Dipping	Freshwater pond/drainage ditch
*Cx. pipiens* (*s.l.*), *Cx. modestus*	Elmley National Nature Reserve, Kent	51°22'59.288"N, 0°46'57.459"E	Larvae, pupae	F0	Dipping	Freshwater pond/drainage ditch
*Cx. pipiens* (*s.l.*)	West Byfleet, Surrey	51°20'35.084"N, 0°29'56.664"W	Eggs	F0	Dipping	Container
*Ae. detritus*, *Ae. caspius*	Wallasea Island, Essex	51°36'55.065"N, 0°49'22.066"E	Adults	F0	CO_2_ traps	Saltmarsh
*Ae. rusticus*, *Ae. cantans*/*Ae. annulipes*	Bartley Heath, Hampshire	51°16'32.568"N, 0°57'18.207"W	Adults	F0	Human landing	Woodland/temporary groundwater
*Ae. detritus*	Dee Marsh, Cheshire	53°16'39.48"N, 3°4'5.286"W	Larvae, pupae	F0	Dipping	Saltmarsh

^a^The Caldbeck colony was established from filial generation ~f50 at APHA in February 2015

^b^The Brookwood colony ~f51 in February 2016

^c^Collected, colonised and characterised by the Pirbright Institute [22]

Dipping refers to the use of pans to collect larvae using a scooping action to target collection of *Aedes* spp. or a tilting action allowing the passive flow of water into the pan to target *Culex* larvae as described previously [50]. The females of *Ae. cantans* and *Ae. annulipes* cannot be reliably identified based on morphological traits [51]. Our attempts to molecularly confirm the identity of specimens from Bartley Heath by using the DNA barcoding retrieved ambiguous results for the species; therefore, we treat *Ae. cantans/Ae. annulipes* in our dataset. Locations are based on survey sites from [22, 52–54], a map of locations is presented in Additional file 1: Figure S1

RVFV strains used to assess vector competence

All manipulations of samples known or suspected to contain infectious RVFV were performed within Biosafety level 3 (BSL-3) laboratories. Two strains of RVFV were used: ZH501 (mouse brain suspension; passage 10) isolated from a fatal human haemorrhagic case in Egypt 1977 [10] and Lunyo (mouse brain suspension; passage 11) isolated in 1953 from *Aedes* mosquitoes in the Lunyo Forest, Uganda [23, 24]. Strain ZH501 was selected as it is a well characterised strain used in over 30 previous studies of vector competence (reviewed by Lumley et al. [5]), the second strain Lunyo was selected to permit comparison to a divergent strain from a different clade [25] with 96.4% homology to ZH501 at the nucleotide level. Virus was propagated on Vero E6 cells at 37 °C for 3 days and quantified by quantitative reverse transcription (qRT)-PCR and plaque assay. Virus was concentrated for use at the higher titre (to account for dilution during blood meal preparation) using 100 kDa AmiconUltra-15 centrifugal filter devices (Merck Millipore, Feltham, UK) at 3,800× *g* for 40 min at 4 °C following manufacturer’s instructions and adapted from [26]. Stocks were stored at -80 °C.

### Evaluating vector competence

**Fig. 1:**
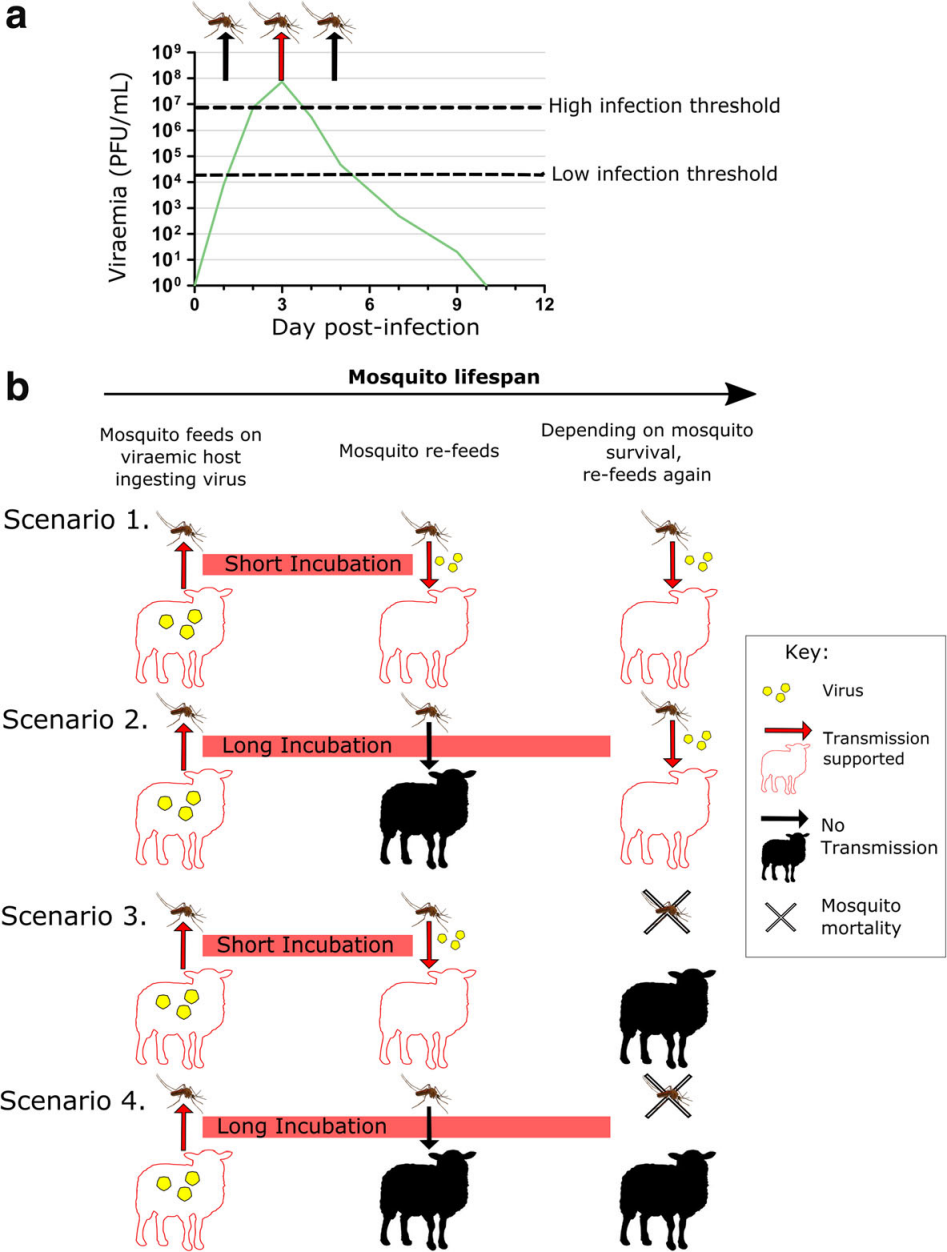
Timeline of events effecting mosquito viral transmission. **a** Schematic of a typical livestock viraemia profile based on experimental infection of ruminants reviewed by Golnar et al. [28]. Viraemia is affected by both age and species of the host, dose, strain and route of inoculum [1], the schematic is therefore representative not absolute. Dotted lines represent a high threshold set by Golnar et al*.* [28] and a low dose threshold required to infect mosquitoes based on the findings of Vloet et al. [44], the latter facilitates onward infection of the mosquito across a longer duration than the high infectious dose [1]. **b** Interplay of the extrinsic incubation period (EIP) and mosquito lifespan on transmission. The EIP is the time from virus ingestion to the first point the mosquito is capable of expectorating virus *via* its saliva during feeding. The shorter the EIP and longer the lifespan the higher the potential for transmission events to occur, both are affected by multiple factors including environment and species specific traits. Scenario 1: short EIP but long lifespan is optimal for a transmission event; Scenario 2: long EIP with a long lifespan; Scenario 3: short EIP with a short lifespan; Scenario 4: long EIP coupled with short a lifespan does not support transmission. Arrows represent a feeding event, with the arrowhead indicating the direction of virus transmission. Red arrows and red sheep: events support transmission; black arrows and black sheep: events do not support transmission

Mosquitoes were offered blood-meals containing either RVFV strain at two doses: 10^6^ and 10^7^ plaque forming units (PFU)/ml which fall within the natural livestock viraemia [27, 28]. A typical viraemia profile for a susceptible vertebrate species previously demonstrating support of onward transmission to vectors is shown in Fig. 1a [28]. Prior to feeding, sucrose was removed and mosquitoes were maintained on water for 16–22 h and starved of water for the last three hours. Mosquitoes (5–10 days-old) were fed through a Parafilm M (Sigma) membrane overnight (15–18 h) in a darkened BugDorm (Bugzarre, Lowestoft, UK), using a hemotek (Hemotek Ltd., Blackburn, UK) set at 37 °C. Infectious RVFV is reported to be highly stable in proteinaceous mediums, preliminary experiments (data not shown) demonstrated a less than one log reduction in infectious RVFV titre in blood maintained at 37 °C for 24 h simulating an artificial blood meal (reviewed by EFSA [29]). The blood meal was prepared using defibrinated horse blood (TCS Biosciences), 1 μM dATP (final concentration) (Thermo Fisher Scientific, Warrington, UK) and RVFV at a ratio of 1:2 (virus: blood). Control blood was prepared substituting virus for Dulbecco’s modified Eagle medium (DMEM) (Thermo Fisher Scientific). Blood-meal samples were taken before and after feeding and stored at -80 °C for quantification.

Post-feeding, mosquitoes were anaesthetised using FlyNap (Carolina Biological Supply, Burlington, US) and engorged mosquitoes sorted into groups of 10–15 in to 73 × 118 mm microhabitat pots with a mesh vent (Bugzarre). Mosquitoes were sampled on day 0 to calculate the dose ingested. Mosquitoes were maintained at 20 or 25 °C on water-soaked cotton and filter paper discs saturated in mānuka honey. Survival was monitored periodically and water replaced as required. Honey-filters were collected every 24–72 h from day 7 onwards and stored at -80 °C. At designated collection points, 7, 14 and 21 days post-infection (dpi), mosquitoes were anaesthetised with FlyNap. Legs and wings were collected, to assess virus dissemination, into 2 ml tubes containing 14 mm ceramic beads (Stretton Scientific, Stretton, UK) and 300 μl mosquito buffer (Eagle’s minimum essential medium supplemented with 20% FBS and 100 units/ml penicillin, 100 μg/ml streptomycin, 0.25 μg/ml amphotericin B) (Sigma-Aldrich). Saliva was collected to assess virus transmission [30]. The proboscis was inserted into a trimmed 200 μl micropipette tip containing 20 μl mosquito buffer supplemented with 50% sucrose. Five microlitres of pilocarpine mix was applied to the abdomen to encourage salivation [1% pilocarpine hydrochlorides (Thermo Fisher Scientific), 0.1% Tween 80 in PBS (Sigma-Aldrich)] and after 30–45 minutes media containing expectorated saliva was expelled into 280 μl mosquito buffer. To reduce PCR inhibition mosquitoes were decapitated [31] and the body (abdomen and thorax) collected and stored as were the legs. Body and legs were disrupted by two 20 s cycles of 4500× *rpm* with a 30 s mid-cycle pause on the Precellys 24 homogeniser (Stretton Scientific). All samples were centrifuged at 3000× *g* for 10 min and processed in the first instance for virus by qRT-PCR. The remaining sample was stored at -80 °C and plaque assays were performed on positive samples only.

### Virus detection and quantification

Viral RNA was extracted using the QIAamp viral RNA mini kit (Qiagen, Manchester, UK) or TRIzol LS kit (Thermo Fisher Scientific) in accordance with the manufacturer’s instructions and eluted in 60 μl. Honey-filters were placed directly into lysis buffer and extracted in parallel with other sample types. Published qRT-PCRs targeting the M and S segments [32, 33] were performed on the Viia 7 real-time PCR system (Thermo Fisher Scientific), using reaction and cycling parameters recommended for the TaqMan Fast Virus 1-step master mix (Thermo Fisher Scientific) with 5 μl template, analysed at baseline 0.05. Genome equivalent copies (GEC) were calculated using the molecular weight and sequence length of a synthetic transcript (GeneArt, Thermo Fisher Scientific). To confirm samples were correctly collected and extracted, published endogenous internal controls were employed targeting host actin for the body and leg samples [34] and mitochondria for saliva samples (an additional reverse primer was designed detailed in Additional file 2: Methods S1) [35]. Samples testing negative for host DNA were removed from the population number for calculations. However, the advent of the saliva host endogenous control PCR came after the 10^6^ PFU dose group experiments were performed and samples were not available to retrospectively test.

Plaque assays were performed on Vero E6 cells (European Collection of Authenticated Cell Cultures), 50 μl of virus dilution was adsorbed for 1 hour at 37 °C. An avicel (Sigma-Aldrich) overlay (0.5 ml 0.6% avicel in MEM) was applied and incubated for 3 days at 37 °C with 5% CO_2_. Overlay for mosquito samples was supplemented with 100 units/ml penicillin, 100 μg/ml streptomycin, 0.25 μg/ml amphotericin B, 100 μg/ml kanamycin, 50 μg/ml gentamicin (Sigma). Cells were fixed and virus inactivated in 10% formaldehyde for 1 h, and stained with 0.2% crystal violet.

### Experimental design

A summary of the experiments performed are shown in Table 2. Colonised mosquitoes were assessed for their competence for RVFV at a constant temperature of 25 °C, corresponding to their routine rearing and maintenance temperature. Wild-caught species were challenged at 25 °C for consistency with colony experiments, and where numbers permitted mosquitoes were also challenged at 20 °C representing peak average temperatures recorded during summer months at larvae collection sites (recorded 1981–2010 by [36]).

**Table 2:** Summary of vector competence experiments performed. The infectious viral titres within the blood meal offered to mosquitoes are written in plaque forming units/ml (PFU)

Mosquito species	10^6^ PFU				10^7^ PFU	
	ZH501		Lunyo		ZH501	Lunyo
*Cx. pipiens* (Caldbeck colony)	✓		–		✓	–
	25 °C				25 °C	
*Cx. pipiens* (Brookwood colony)	–		✓		✓	✓
			25 °C		25 °C	25 °C
*Ae. detritus* (Wild-caught)	✓		✓		✓	✓
	20 °C	25 °C	20°C	25 °C	20 °C	20 °C
*Ae. rusticus* (Wild-caught)	✓		–		✓	–
	25 °C				20 °C	

✓ Vector competence experiment was performed under the conditions listed with the incubation temperature written below

– denotes experiment condition not tested

### Statistical analyses

Infection, dissemination and transmission rates were calculated as the percentage of blood feeding mosquitoes that survived until a collection point (and positive for endogenous PCR), containing virus in their bodies, legs and saliva, respectively. Sample numbers were guided by the herd sensitivity model for an infinite population size, signifying a sample size requirement of 29 to detect a single positive specimen from a population with 10% transmission capability with 95% confidence [37]. Doses and virus strains were compared by Fisher’s exact test, using significance threshold 0.05 with Bonferroni-corrected thresholds for multiple comparisons. 95% confidence intervals (95% CI) were calculated by a modified Wald method [38]. Titres were compared using Mann-Whitney or Kruskal-Wallis tests followed by Dunn’s *post-hoc* tests for multiple comparisons. Survival was analysed by a Kaplan-Meier plot (GraphPad Prism), mosquitoes processed for virus detection were censored and groups compared by Log-Rank test.

## Results

### Mosquito survival

**Fig. 2:**
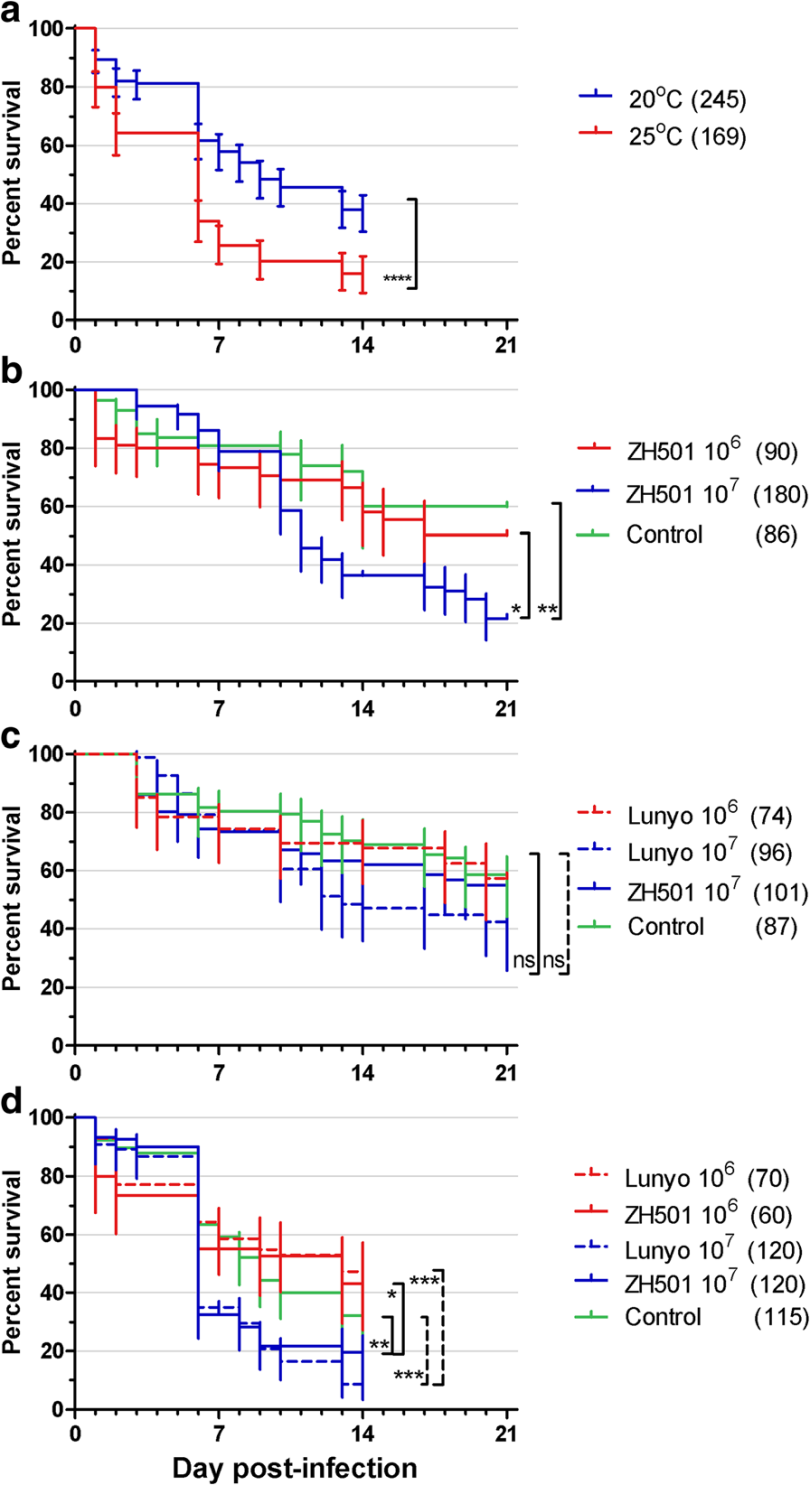
Percentage survival of mosquitoes. **a** Effect of temperature on *Ae. detritus* survival. Mosquitos ingesting a blood meal containing at 10^6^ PFU/mL RVFV strain ZH501, Lunyo or an uninfected control were maintained at 20 or 25 °C. Survival did not differ statistically between strains or controls at 20 °C (Log-rank (Mantel-Cox) *χ*^2^ = 1.174, *df* = 2, *P* = 0.5560) or at 25 °C (*χ*^2^ = 0.9600, *df* = 2, *P* = 0.9600) so datasets were pooled for analysis, demonstrating reduced survival at 25 °C compared to 20 °C. The effects of viral dose on survival are presented in panels **b**-**d** comparing 10^6^ PFU/ml *vs* 10^7^ PFU/ml *vs* uninfected controls. **b** *Cx. pipiens* f. *pipiens* (Caldbeck). **c** *Cx. pipiens* hybrid (Brookwood). **d** *Ae. detritus*. Survival was monitored periodically for up to 21 days. Kaplan-Meier plots were generated in GraphPad Prism, bars represent the 95% confidence interval, the numbers tested are written within parentheses (*n*). Statistical differences were calculated by Log-Rank test with Bonferroni-corrected thresholds, dotted lines compare strain: Lunyo, solid lines: ZH501. Demonstrating reduced survival for *Cx. pipiens* Caldbeck and *Ae. detritus* after ingestion of blood containing 10^7^ PFU/ml compared to controls and their lower dose counterparts but no differences between doses or controls ingested by the Brookwood line mosquitoes

The effects of temperature, RVFV strain and dose on mosquito survival were investigated. Results in Fig. 2a show that *Ae. detritus* survival was significantly reduced at 25 °C under experimental conditions compared to 20 °C when monitored over 14 days (Log-rank (Mantel-Cox) test: *χ*^2^ = 38.06, *df* = 1, *P* < 0.0001). Results for both RVFV strains and controls were combined (Fig. 2a) as the effect of temperature on survival at 25 °C compared to 20 °C appeared independent of virus infection. Significantly more *Ae. detritus* mosquito survived when infected with 10^6^ PFU/ml ZH501 (Log-rank (Mantel-Cox) test: *χ*^2^ = 12.28, *df* = 1, *P* = 0.0005), Lunyo (*χ*^2^ = 14.03, *df* = 1, *P* = 0.0002) and the uninfected controls (*χ*^2^ = 6.042, *df* = 1, *P* = 0.0140). Repetitions of experiments using 10^7^ PFU/ml were therefore performed at 20 °C to promote survival and increase experimental numbers to evaluate competency. Reduced survival at 25 °C was also observed with *Ae. rusticus*; however sample numbers were too small to perform statistical analysis with just 2 of 20 surviving until 7 dpi.

The effect of RVFV strain on mosquitoes at equivalent doses and temperatures, did not differ significantly between strains (Fig. 2b-d). It was observed that when a higher dose of virus was ingested, mortality increased. A significant increase in *Ae. detritus* mortality was observed following infection with both RVFV strains (Log-rank (Mantel-Cox) test: ZH501 *χ*^2^ = 4.362, *df* = 1, *P* = 0.0367; Lunyo *χ*^2^ = 18.888, *df* = 1, *P* <0.0001) (Fig. 2d) and in *Cx. pipiens* (CBK) infected with strain ZH501 (*χ*^2^ = 6.607, *df* = 1, *P* = 0.0102) (Fig. 2b) when compared to uninfected controls and lower dose counterparts. Mortality rates between virus doses in the Brookwood line were not statistically different (ZH501 *χ*^2^ = 0.2687, *df* = 1, *P* = 0.6042; Lunyo *χ*^2^ = 2.948, *df* = 1, *P* = 0.0860) (Fig. 2c).

### Vector competence

The mean titres of RVFV in the blood meal before and after feeding mosquitoes in the low dose group was 10^6.0^ and in the higher dose group was 10^7.2^ PFU/ml, decreasing on average by 0.26-logs during overnight feeding. Data for individual experimental groups are presented in Additional file 3: Figure S2. Reduction in virus titre in the mosquito compared to the blood meal indicated mosquitoes ingested between 1–10 μl of blood. Based on a measurement of GECs, levels of RVFV in engorged mosquitoes were similar irrespective of the mosquito species (Additional file 3: Figure S2).

**Fig. 3:**
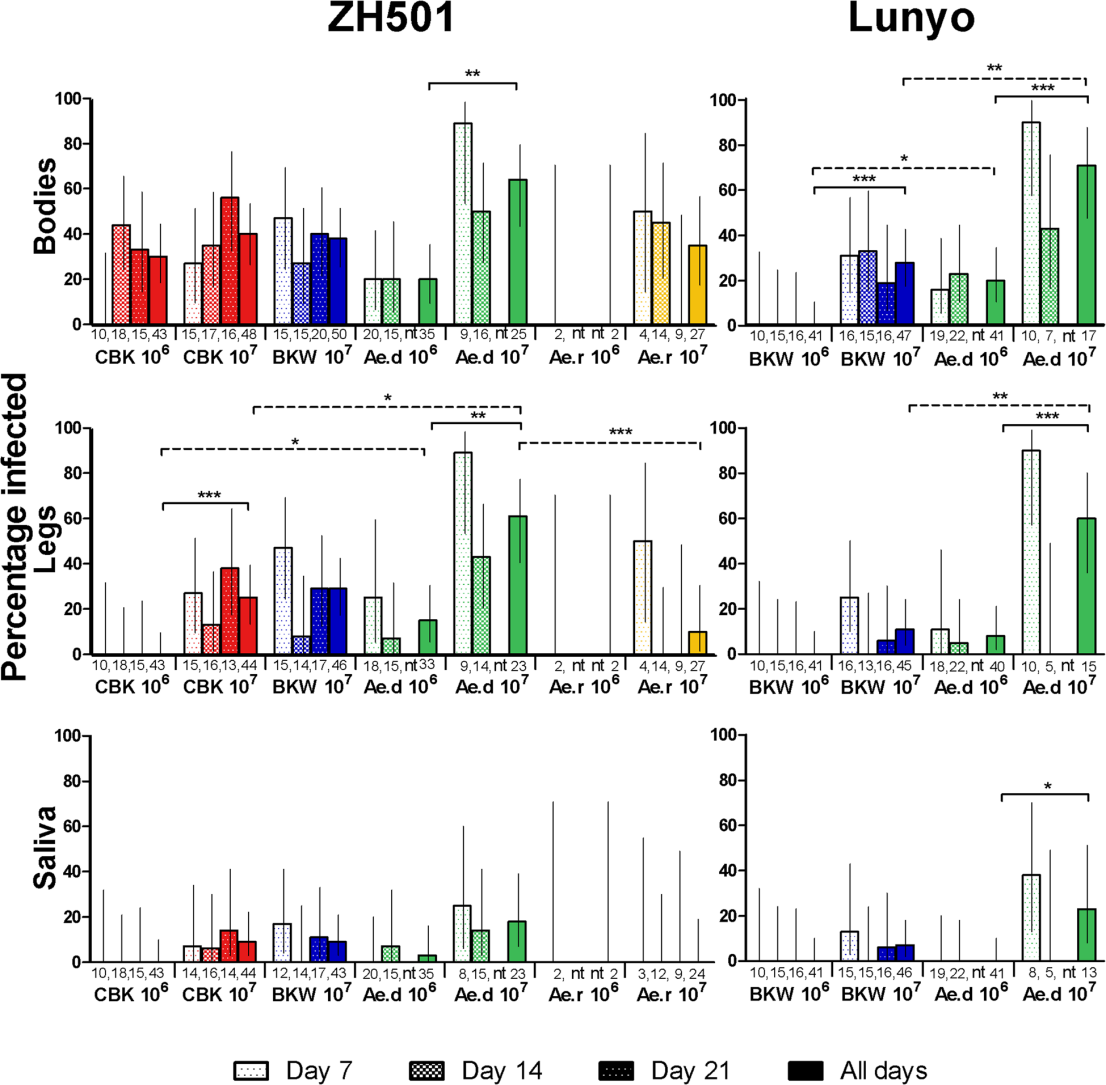
Percentage of mosquitoes infected with Rift Valley fever virus. Mosquitoes were fed a blood-meal containing 10^6^ or 10^7^ PFU/ml Rift Valley fever virus strain Lunyo or ZH501 and maintained at 20 or 25 °C. Results are based on the proportion of mosquitoes positive for RVFV by qRT-PCR results. Day 0 was excluded from the totals. Numbers below bars represent the total number tested; error bars indicate 95% confidence intervals calculated by a modified Wald method. Fisher’s exact test was used to compare between time points, doses and virus strains using a Bonferroni-corrected threshold for multiple comparisons; comparisons between mosquito species are depicted by dotted bars, and comparison of doses by solid bars. *Abbreviations*: nt, not tested; CBK, *Cx. pipiens* (Caldbeck); BKW, *Cx. pipiens* (Brookwood); Ae.d, *Ae. detritus*; Ae.r, *Ae. rusticus*

The proportion of mosquitoes demonstrating infection, dissemination from the midgut, and a transmission potential for RVFV determined by qRT-PCR are shown in Fig. 3. Infectious viral particles determined by plaque assay were detected in the body and leg samples but not the saliva, with qRT-PCR results suggesting that viral titres in saliva were below the limit of detection of our plaque assay (Additional file 4: Table S1). The virus strain used had no significant effect on infection, dissemination or transmission rates in any of the mosquito species challenged with either RVFV strain; *Ae. detritus* (Fisher’s exact test: bodies *P* = 1.0000; legs *P* = 0.6870; saliva *P* = 1.0000) and *Cx. pipiens* (BKW) (Fisher’s exact test: bodies *P* = 0.2910; legs *P* = 0.1697; saliva *P* = 1.0000).

Virus dose had the greatest impact on outcomes with all species showing an increase in infection, dissemination or transmission rate when mosquitoes were infected with the higher titre of virus. Due to low sample numbers, rates in *Ae. rusticus* were not compared. Rates of infection were significantly higher for all groups except *Cx. pipiens* (CBK) and dissemination for all except the *Cx. pipiens* (BKW) infected with RVFV strain Lunyo. The transmission rates for mosquitoes infected with 10^7^ PFU (or 10^6^ PFU reported in parentheses) were: 9% (0%) for *Cx. pipiens* (CBK) infected with strain ZH501; 9 and 7% (0%) for BKW infected with ZH501 and Lunyo; 18% (3%) and 23% (0%) for *Ae. detritus* strain ZH501 and Lunyo; and 0% (0%) for *Ae. rusticus* with ZH501. However, despite these observed increases, only the transmission rate by *Ae. detritus* infected with strain Lunyo increased significantly at the higher dose (Fisher’s exact test: *P* = 0.0115). Only transmission rates between species *Ae. detritus* and *Ae. rusticus* infected with 10^7^ PFU ZH501 differed significantly (Fisher’s exact test: *P* = 0.0345).

Quantification of viral titres within different mosquito tissues can be used to indicate replication and evaluate the potential dose dependency of barriers encountered by the virus whilst progressing through the mosquito. The titres observed within mosquito bodies were fairly uniform across conditions and did not differ statistically between strains or the dose ingested (Mann-Whitney U-test: *P* ≥ 0.05) (Additional file 4: Table S1). Temporal collection of honey-filters from groups of 10–15 mosquitoes corroborated the results of individual mosquito salivation, with RVFV RNA detected in the saliva of both *Cx. pipiens* lines and *Ae. detritus* by day 7 post-infection, but not from *Ae. rusticus*. The average viral titres detected in the saliva were 10^2.8^ GEC ZH501 and 10^3.6^ GEC Lunyo and did not differ between mosquito species (strain ZH501 Kruskal-Wallis test: *H* = 1.196, *P* = 0.5498, strain Lunyo Mann-Whitney test: *U* = 2.00, *P* = 0.4000) or collection method for *Cx. pipiens* (CBK) (Mann-Whitney test: strain ZH501 *U* = 17.00, *P* = 0.2648), *Cx. pipiens* (BKW) (ZH501 *U* = 4.00, *P* = 0.3429; Lunyo *U* = 13.00, *P* =1.0000) or *Ae. detritus* (ZH501 *U* = 26.00, *P* = 0.1580; Lunyo *U* = 16.00, *P* = 0.8286) (Additional file 4: Table S1).

In addition to competence experiments performed with wild-caught *Ae. detritus* and *Ae. rusticus,* further wild-caught species were investigated (Table 1). *Aedes cantans/annulipes* collected from woodlands were not susceptible to infection; however, the sample size was small (*n* = 7). *Culex pipiens* were collected from three locations in southern England but failed to feed using an artificial feeding device. *Culex modestus* and *Ae. caspius* were collected from sites with mixed populations. Although sample numbers were low, at 10 dpi a single *Cx. modestus* had disseminated virus (1 of 5) and a single *Ae. caspius* had RVFV RNA in the saliva (1 of 6).

## Discussion

This study assessed British populations of mosquitoes for their ability to support and transmit RVFV. Under laboratory conditions, two lines of *Cx. pipiens* and *Ae. detritus* demonstrated the potential to transmit RVFV, with the detection of low levels of viral RNA in expectorated saliva. There were no differences in the rates of mortality, infection, dissemination or transmission between strains ZH501 or Lunyo but mosquito survival was negatively affected by high viral dose (10^7^ PFU) and at the higher temperature (25 °C).

Ingested viral dose had a significant effect on the infection, dissemination and transmission rates, concurring with previous studies [28, 39, 40]. Natural peak viraemia in calves and lambs range between 10^6^–10^8^ PFU/ml [27, 28]. Two doses at the low (10^6^ PFU) and medium (10^7^ PFU) range of this viraemia were evaluated to determine the lower threshold required to artificially infect mosquitoes. Neither of the *Cx. pipiens* lines were able to support dissemination of virus from the midgut at the lower dose and therefore could not transmit RVFV. At this lower dose the *Cx. pipiens* Caldbeck line, supported replication but the Brookwood line did not, suggesting the presence of a barrier to escape from and infection of the midgut, respectively in these mosquito lines. Multiple mechanisms have been postulated, including physical barriers to virus or incompatibility with host receptors or antiviral responses including RNA interference and degradation by midgut enzymes [41]. Further work is required to understand the mechanisms involved for these species, for example using microscopy to evaluate virus localisation. However, *Ae. detritus*, once infected with RVFV, supported high proportions of disseminated infection at the lower dose and virus was detected in saliva of a single mosquito demonstrating that this species has the potential to transmit RVFV.

Combining the collection points at the low dose we analysed 43 (ZH501) *Cx. pipiens* (CBK), 41 (Lunyo) *Cx. pipiens* (BKW) and 35 (ZH501) and 36 (Lunyo) *Ae. detritus*. Assuming less than 10% of mosquitoes within a population can transmit, the number of test specimens required to detect at least one positive with 95% confidence is 29 mosquitoes (herd sensitivity model, assuming infinite population size [37]). Although greater numbers would increase the statistical power, these data support this inability to transmit virus where results were negative, based on the numbers evaluated.

Transmission increased in all species at the medium dose of 10^7^ PFU, except *Ae. rusticus* which demonstrated limited capability to disseminate at later time points; however, the low sample numbers (*n* = 20) for this species limit the strength of this observation. At this dose the barriers to infection were generally overcome in both *Culex* lines; virus infected a moderate number and escaped the midgut of a large proportion of infected specimens. *Aedes detritus* had high infection and dissemination rates but the mosquitoes did not differ in their transmission rates compared to *Culex*, suggestive of potential salivary gland barriers as observed previously by Gargan et al. [42]. Further work is needed to confirm this hypothesis, and the methods used cannot differentiate between an infection or escape barrier; dissecting the salivary glands and detailed microscopy could increase our understanding of the underlying mechanisms [41].

Viral titres in the saliva did not differ as a result of mosquito species, infectious dose or RVFV strain (Additional file 4: Table S1). Assessing the potential to transmit based on levels of RVFV RNA in saliva, suggests that there is an equal capability between species to pass virus horizontally to vertebrates, although this observation is limited by the low number of mosquitoes expectorating virus. Failure to detect infectious virus in the saliva is likely a limitation of the plaque assay technique. Diluting the samples to screen by sensitive molecular methods increased throughput but introduced a freeze-thaw potentiating virus degradation. Although the detection of viral RNA in saliva demonstrates a potential for transmission, it is not known if the titres detected here are sufficient to cause disease. Previous reports have suggested that *in vitro* collection of saliva underestimates titres and local inflammatory responses in the vertebrate may facilitate viral infection [43, 44]. Therefore, important next steps are demonstrating this ability to transmit functional virions to an animal model resulting in disease, to confirm transmission.

The dose dependence observed limits the period that feeding mosquitoes could support transmission in the field, to the short duration of the peak viraemia (Fig. 1a) as reported previously [44]. This peak viraemia typically occurs on day three post-infection in young ruminants and lasts for less than 24 h. Based on data presented using artificial blood meals, mosquitoes feeding outside of this window would not transmit RVFV, although previous observations suggest infection and transmission rates may be underestimated and dose threshold overestimated by the use of artificial blood meals compared to feeding on a viraemic hamster or lamb [44, 45]. It is feasible, therefore, that these differences in experimental design account for the lower rate of transmission observed in *Cx. pipiens* derived from the UK compared to the Netherlands [44] rather than genetic differences between different populations of the same species [15].

The temporal interplay between peak viraemia of the vertebrate host and progression of virus to the saliva of the vector host are important, fundamentally supporting or prohibiting transmission (Fig. 1). The extrinsic incubation period for transmitting mosquitoes was ≤ 7 days post-infection, however earlier time points were not tested. Although the durations between seeking blood meals are unknown for these species, the ability to transmit within just 7 days of ingestion of viruses increases the transmission potential in subsequent feeds (Fig. 1b, scenarios 1 and 3). Survival of the virus, and ultimately transmission, is reliant on vector host survival. Differences in mosquito mortality rates are likely to have the greatest impact on the capacity to transmit virus in the field (Fig. 1b, scenario 3). High mosquito mortality was observed at 25 °C compared to 20 °C in field-caught *Aedes* species; the concurrent mortality of controls and infected mosquitoes suggests a virus-independent factor. It is conceivable that both *Aedes* populations have adapted to cooler (temperate) climates in their coastal and woodland habitats. The high mortality observed at 25 °C, limited the ability to directly compare vector competence at two temperatures applied to the wild-caught species in this study. Although temperature increases are typically observed to increase vector competence, similar findings of an inverse relationship with temperature and vector capacity have been observed previously with increased mortality limiting transmission [46]. However, the effect of natural diurnal temperature fluctuations should also be considered [46] and the lower thermal limit at which RVFV transmission can occur determined.

Increased mortality in *Cx. pipiens* Caldbeck and *Ae. detritus* infected with the higher viral dose was also observed, consistent with previous reports [47]. This virus-dependent factor occurred exclusively in infected (not control) groups. Despite high disseminated titres in the hybrid, survival did not differ significantly between dose groups, thereby increasing its capacity as a vector. Tissue damage and resource depletion as a result of rapid viral amplification have been postulated as causes of cytopathic effects observed in flavivirus-infected mosquitoes [48]. Further studies comparing the high and low mortality groups observed could advance our understanding of mechanisms affecting survival.

Multiple factors including changes in land-use and climate predict increases in mosquito species and numbers within Europe, thereby increasing the risk of arbovirus emergence and establishment [49]. Despite the limitation of studying mosquitoes under experimental conditions, this approach offers important data for identifying temperate mosquitoes present in the UK as potential vectors of RVFV.

## Conclusions

The spread of RVFV to Europe and its establishment would severely impact the health of humans, livestock and the economy. To assess the risk of establishment, an understanding of the ability of indigenous mosquitoes to support and transmit RVFV is required. However, vector competence remains one element of vector capacity so it is important to pair these data with behavioural and life-history traits in the field, such as host-feeding preference, time between feeding events and survival. We report the ability of laboratory colonised populations of *Cx. pipiens* and wild-caught *Ae. detritus* from the UK to transmit RVFV RNA in their saliva. These data suggest that comparable wild mosquito species surviving under parallel environmental conditions might also have the ability to transmit RVFV. In these natural habitats and based on the feeding preferences of *Ae. detritus*, it can be further speculated that under the right circumstances these temperate mosquito species, that are competent for RVFV, may be able to infect susceptible livestock and pose a threat to public health.

## Additional files

Additional file 1: **Figure S1.** Map of mosquito collection site locations. Map generated using ArcMap version 10.4. (TIF 70791 kb)

Additional file 2: **Methods S1.** Supplementary methods: endogenous PCR control for mosquito saliva samples. (DOCX 27 kb)

Additional file 3: **Figure S2.** Viral titres within the blood meal and ingested by mosquitoes. Dots represent median RVFV titres per ml of blood, quantified before and after feeding. Boxes represent the RVFV titres ingested by mosquitoes, horizontal lines at the median and whiskers showing minimum and maximum values. Samples were stored at -80 °C. Genome equivalent copies (GEC) and infectious virus titres (PFU) were calculated by qRT-PCR and plaque assay. *Abbreviations*: CBK, *Cx. pipiens* (Caldbeck colony); BKW, *Cx. pipiens* (Brookwood colony); Ae.d, *Ae. detritus*; Ae.r, *Ae. rusticus*. Statistical comparison of ingested dose (GEC) was performed by Mann-Whitney U-test in GraphPad prism: Lunyo *Cx. pipiens* Brookwood *vs Ae. detritus* 10^6^ dose cohort: *U* = 18.00, = 0.171; 10^7^ cohort: *U* = 28.00, *P* = 0.1040; ZH501 dose 10^6^ *Cx. pipiens* Caldbeck *vs Ae. detritus*: *U* = 86, *P* = 0.5524; and all four mosquito populations infected with ZH501 at 10^7^ Kruskal-Wallis test: *H* = 0.5458, *P* = 0.9087). Demonstrating uniformity between the experimental groups and mosquito species. (TIF 190 kb)

Additional file 4: **Table S1.** Viral RNA and infectious viral titres within the bodies, legs and saliva of UK mosquitoes infected with RVFV. Mosquitoes were fed a blood meal containing 10^6^ or 10^7^ PFU/ml RVFV strain Lunyo or ZH501 and maintained at 20 or 25 °C. Results are based on the mean log_10_-transformed qRT-PCR results reported in genome equivalent copies (GEC) and plaque assay results reported in plaque forming units (PFU). Day 0 was excluded from the totals. *Abbreviations*: nt, not tested; *n*, number tested; dpi, day post-infection. Dose range based on infectious titres obtained in pre and post feed blood samples. (XLSX 18 kb)
