# Supplementary material for: Competence of mosquitoes native to the United Kingdom to support replication and transmission of Rift Valley fever virus
Source: Parasit Vectors. 2018 May 18;11:308. doi: 10.1186/s13071-018-2884-7 (PMC5960175; doi:10.1186/s13071-018-2884-7)
Supplement: Supplementary file 2 — Methods S1. Supplementary methods: endogenous PCR control for mosquito saliva samples. (DOCX 27 kb) [file 13071_2018_2884_MOESM2_ESM.docx]

A mosquito endogenous control PCRs was utilised targeting host mitochondrial DNA for saliva. The PCR was performed on all saliva samples in the higher dose experiments (10^7^ PFU), performed on the 7500 Fast Real-Time PCR System (Thermo Fisher Scientific). Grubaugh *et al*. (2017) developed the PCR using sequences derived from *Cx. quinquefasciatus* stating its scope for use for targeting samples derived from *Cx.* spp. only. Here, an additional reverse primer (Saliva Rev2) was designed based on the sequence alignment results shown below. Saliva Fwd :3’-TTCTGATGACGGCGATATACAAATT-5’, Saliva Rev1: 3’- GGCGGTATTTTAGTCTATTCAGAGGA-5’, Saliva Rev2: 3’- GGCGGTGTTTTAGTCTATTTAGAGGA-5’. The PCR amplified both *Aedes* and *Culex* species used within these experiments. Master mix composition and cycling parameters were setup as recommended for the kit. Assays were performed using the iTaq Universal SYBR Green one-step kit (Bio-Rad): 2.75µL water, 10 µL iTaq SYBR reaction mix, 1 µL saliva fwd (10µM), 0.5 µL saliva rev1 (10µM), 0.5 µL saliva rev2 (10µM), 0.25µL iScript RT and 5µL template. Cycling: 50^o^C for 10 minutes (1 cycle); 95^o^C for 1 minute (1 cycle); 95^o^C for 15 seconds and 60^o^C for 30 seconds (40 cycles) with quantification of fluorescence assessed at the end of each 60^o^C step and melt curve analysis at the end of run using standard settings on the 7500 Fast Real-Time PCR System (Thermo Fisher Scientific).

Sequence alignment of saliva PCR oligonucleotides to *Culex* and *Aedes* species.

Alignment of accession numbers: MF194022, KR068634, KM676219, KP995260, EF028703, HQ724615, HQ724616, KT851543, KT851544, MF040162, MF040164 were performed in MegAlign, DNAStar. Homologous sites are represented by dots and mismatches denoted by the variant nucleotide letter.
