# Supplementary figures and images for: Competence of mosquitoes native to the United Kingdom to support replication and transmission of Rift Valley fever virus
Source: Parasit Vectors. 2018 May 18;11:308. doi: 10.1186/s13071-018-2884-7 (PMC5960175; doi:10.1186/s13071-018-2884-7)

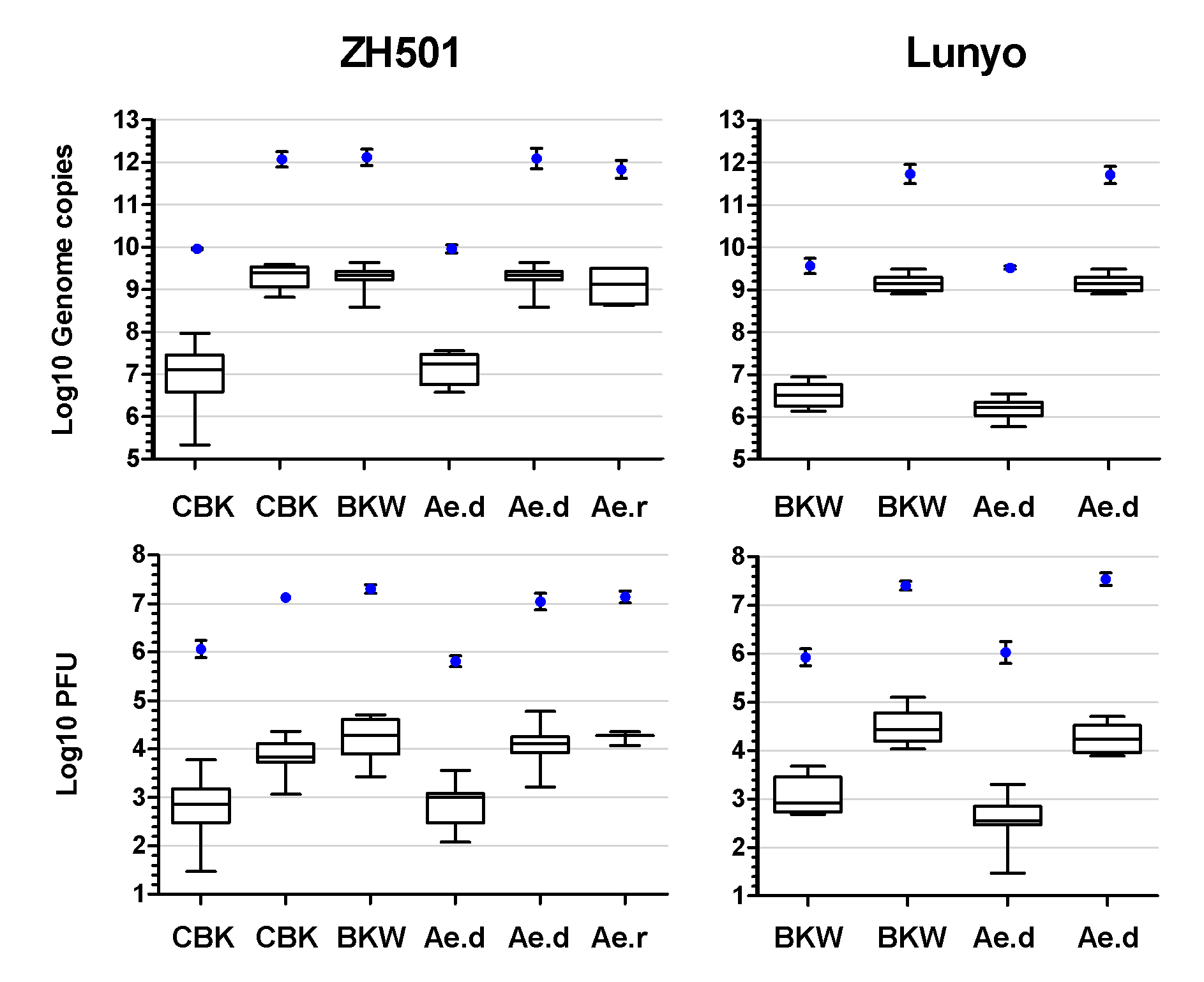

Supplement: Supplementary file 3 — Figure S2. Viral titres within the blood meal and ingested by mosquitoes. Dots represent median RVFV titres per ml of blood, quantified before and after feeding. Boxes represent the RVFV titres ingested by mosquitoes, horizontal lines at the median and whiskers showing minimum and maximum values. Samples were stored at -80 °C. Genome equivalent copies (GEC) and infectious virus titres (PFU) were calculated by qRT-PCR and plaque assay. Abbreviations: CBK, Cx. pipiens (Caldbeck colony); BKW, Cx. pipiens (Brookwood colony); Ae.d, Ae. detritus; Ae.r, Ae. rusticus. Statistical comparison of ingested dose (GEC) was performed by Mann-Whitney U-test in GraphPad prism: Lunyo Cx. pipiens Brookwood vs Ae. detritus 106 dose cohort: U = 18.00, = 0.171; 107 cohort: U = 28.00, P = 0.1040; ZH501 dose 106 Cx. pipiens Caldbeck vs Ae. detritus: U = 86, P = 0.5524; and all four mosquito populations infected with ZH501 at 107 Kruskal-Wallis test: H = 0.5458, P = 0.9087). Demonstrating uniformity between the experimental groups and mosquito species. (TIF 190 kb) [file 13071_2018_2884_MOESM3_ESM.tif]
